# Microbiome Data Accurately Predicts the Postmortem Interval Using Random Forest Regression Models

**DOI:** 10.3390/genes9020104

**Published:** 2018-02-16

**Authors:** Aeriel Belk, Zhenjiang Zech Xu, David O. Carter, Aaron Lynne, Sibyl Bucheli, Rob Knight, Jessica L. Metcalf

**Affiliations:** 1 Department of Animal Sciences, Colorado State University, Fort Collins, CO 80525, USA; aeriel.belk@colostate.edu; 2 Department of Pediatrics, University of California, La Jolla, San Diego, CA 92093, USA; zhx054@ucsd.edu (Z.Z.X.); robknight@ucsd.edu (R.K.); 3 Laboratory of Forensic Taphonomy, Forensic Sciences Unit, Division of Natural Sciences and Mathematics, Chaminade University of Honolulu, Honolulu, HI 96816, USA; david.carter@chaminade.edu; 4 Department of Biological Sciences, Sam Houston State University, Huntsville, TX 77340, USA; aml027@shsu.edu (A.L.); srb009@shsu.edu (S.B.); 5 Department of Computer Science and Engineering, University of California, San Diego, CA 92037, USA;; 6 Microbiome Innovation Center, University of California, San Diego, CA 92037, USA

**Keywords:** postmortem interval, microbiome, decomposition, Random Forest regression

## Abstract

Death investigations often include an effort to establish the postmortem interval (PMI) in cases in which the time of death is uncertain. The postmortem interval can lead to the identification of the deceased and the validation of witness statements and suspect alibis. Recent research has demonstrated that microbes provide an accurate clock that starts at death and relies on ecological change in the microbial communities that normally inhabit a body and its surrounding environment. Here, we explore how to build the most robust Random Forest regression models for prediction of PMI by testing models built on different sample types (gravesoil, skin of the torso, skin of the head), gene markers (16S ribosomal RNA (rRNA), 18S rRNA, internal transcribed spacer regions (ITS)), and taxonomic levels (sequence variants, species, genus, etc.). We also tested whether particular suites of indicator microbes were informative across different datasets. Generally, results indicate that the most accurate models for predicting PMI were built using gravesoil and skin data using the 16S rRNA genetic marker at the taxonomic level of phyla. Additionally, several phyla consistently contributed highly to model accuracy and may be candidate indicators of PMI.

## 1. Introduction

Unattended death scenes pose challenges for crime scene investigators because the time of death, also known as the postmortem interval (PMI), is often unknown. However, no death scene is really unattended—microorganisms are ubiquitous, and these tiny witnesses can provide clues about the events surrounding death. For example, communities of microorganisms often have predictable ecologies, which can be leveraged for temporal [1,2] and geographic information [3]. Due to the rapidly decreasing costs of next-generation sequencing, it is feasible and cost-effective to track microbial community change during decomposition via standard microbiome sequencing protocols [4]. Three taxonomically-informative genomic markers—16S ribosomal RNA (rRNA) (archaea and bacteria), 18S rRNA (microbial eukaryotes), and internal transcribed spacer regions (ITS; fungi specifically)—have been widely utilized to characterize microbial community composition and diversity [5,6,7]. Using these markers, recent research has revealed that tracking microbial community succession associated with mammalian cadaver decomposition can be a useful tool for estimating PMI [8]. This idea is very similar to tools developed in the field of forensic entomology, in which the succession of insects can be informative about the time frame and season of death [9]. Several studies have demonstrated consistent changes in microbial community composition during mammalian decomposition associated with skin [10,11,12], gastrointestinal/rectal locations [10,11,13,14], oral sites [12], nasal and ear cavities [15], and cadaver-associated soils [10,11,16,17,18]. These studies have used a variety of model-based statistical approaches for estimating PMI. For example, Pechal et al. [12], utilized an indicator species analysis at the bacterial family taxonomic level over 5 days of decomposition. Furthermore, Hauther et al. [13] utilized an exponential decay model based on declines in relative abundance of particular bacteria such as *Bacteroides*, *Lactobacillus*, and *Bifidobacterium*. However, the most accurate estimates of PMI have employed machine learning approaches [10,11,15], which are ideal for constructing models that utilize changes in relative abundance of all microorganisms in the entire community, as opposed to focusing on a subset of taxa that may not be the most temporally informative.

The reproducibility of microbial community succession during mammalian decomposition indicates that it can be used to predict PMI. However, there is no single microbial species informative enough for accurate prediction. Machine learning is a powerful tool to discover the patterns in complex data and thus can be applied in this case to predict PMI utilizing a diverse microbial community [19]. Using the quantification of each microbial taxa by a marker gene (16S rRNA, 18S rRNA, or ITS) as a predictive feature, supervised regression models can be trained to learn the implicit relationship between microbiome composition and decomposition time point. The Random Forest regression model is widely used because of its robustness to overfitting, excellent performance, and easy parallelization of computing [20]. Random Forest is an ensemble machine learning method that fits a set of decision trees on subsamples of the data set, and then combines the results to improve regression accuracy. Like all tree-based regression methods, Random Forest tends to overestimate the PMI of samples at the low end of PMI and underestimate at the high end of PMI. However, this systematic bias in Random Forest models is well known and can be calibrated with additional data sets [21]. In previous reports, Random Forest regression has been shown to achieve accurate PMI prediction in multiple skin and cadaver-associated soil gene marker data sets across decomposition of different host species [10,11].

We identified several knowledge gaps for developing robust machine learning Random Forest regression models for estimating PMI and addressed them using a meta-analysis of four previously published mammalian decomposition time-series data sets [10,11]. We aim to address which sample type(s), gene marker(s), and taxonomic level(s) provide the most accurate microbial model for estimating PMI. Additionally, we investigated whether particular microbes are informative across different sample types and data sets, which provide insights into whether suites of microbes or microbial groups can be used as indicators, or whether the full community provides the most accurate information. We chose four data sets that represented a range of environments and were generated using a standardized set of microbiome protocols [4]. which makes them directly comparable. We focus our investigations on swabs of skin and gravesoils because these sampling locations would minimally impact the cadaver compared to other, more invasive, locations such as the gastrointestinal (GI) tract. Thus, skin and soils are realistic sample types for development into a viable forensic tool. Each data set included 16S rRNA and 18S rRNA data, and three of the data sets also included ITS data. We looked for consistent trends across the datasets to help point researchers in the most fruitful future directions.

## 2. Materials and Methods

### 2.1. Amplicon Sequencing Data Processing

Previously, published 16S rRNA, 18S rRNA, and ITS data were obtained from the QIITA open-source microbiome study management platform, under studies 714, 1889, 10,141, 10,142, and 10,143 [10,11,22]. Briefly, these studies included two laboratory decomposition experiments [10,11], in which mice were decomposed on soils with the exclusion of insects and destructively sampled in replicates of 5 for 8-time points over 2–3 months. We also included two experiments in which two human donors were allowed to decompose outdoors in the winter season and in the spring season (a total of four donors) at the Southeast Texas Applied Forensic Science (STAFS) laboratory [11]. For further details on the data used for this study, see Table 1. Briefly, for each gene marker in these data sets, the Earth Microbiome Project primer pair and standard protocols were utilized [4]. Amplicons for each gene marker were then sequenced using the Illumina Hiseq 2000 (Illumina, San Diego, CA, USA) platform (2 × 100 bp reads), and forward reads for each gene marker were used to create a feature table of sequences. Sequence data, metadata, and feature tables are available and curated on QIITA where they are periodically re-annotated to be consistent with current best practices utilizing the QIIME pipeline [22,23]. Therefore, we utilized data processed using the deblur method, which utilizes sequence error profiles to derive putatively true biological sequences, resulting in high quality sequence variant data as opposed to operational taxonomic units (OTUs) in which sequence variation is lost because sequences are collapsed, usually at a sequence identity of 97% [24]. In the original publication of these datasets [10,11], a closed reference OTU-picking method was used to generate OTU tables, which likely resulted in the loss of potentially useful sequence data that did not match a reference database [25] within 97% similarity. Therefore, the current meta-analysis provides an opportunity to re-analyze these valuable datasets with more current methods. The 16S rRNA and 18S rRNA amplicon sequence files were trimmed for quality to 90 bp reads. For ITS data, 100bp reads were utilized. For 16S rRNA, the resulting feature table was further processed by removing sequences that did not match a positive reference database with 80% similarity (reference-hit.biom table downloaded). For 18S rRNA and ITS, a positive reference database was not used (all.biom table downloaded). Internal transcribed spacer regions data were only available for studies 10,141, 10,142, and 10,143. For each data set, we retained common sample types, including those taken from gravesoil near the torso, and from the skin of the left hip, right hip, torso, and head. These were categorized into three sample groups for analysis: cadaver-associated gravesoil, skin of the torso, and skin of the head.

### 2.2. Assigning Taxonomy

Each table was individually processed to assign taxonomy and filter out taxa that were not considered part of the microbiome. Taxa were assigned using classifiers specific to each marker: Greengenes 13.8 for 16S rRNA [25], SILVA 128 for 18S rRNA [26], and UNITE 7 developer classifier for ITS [27], all at the 99% sequence identity threshold level. Sequences filtered out of the 16S rRNA data set included those assigned to chloroplasts and mitochondria. Sequences filtered out of the 18S rRNA data included those assigned to Archaeplastida, Arthropoda, Chordata, Mollusca, as well as sequences that were not assigned to Eukarya. For ITS, sequences that did not assign to Fungi were filtered out. Following this, filtered tables were combined into a single sequence variant table per marker type to be used in modeling. These tables were then used to generate additional tables summarized at different taxonomic levels (L), including species (16S rRNA L6; 18S rRNA L12; ITS L6), genus (16S rRNA L5; 18S rRNA L10; ITS L5), family (16S rRNA L4; 18S rRNA L8; ITS L4), order (16S rRNA L3; 18S rRNA L6; ITS L3), class (16S rRNA L3; 18S rRNA L4; ITS L3), and phylum (16S rRNA L2; 18S rRNA L3; ITS L2). SILVA taxonomy levels were very uneven across different groups of eukaryotes (e.g., Amoebozoa, Opisthokonta, and Alveolata), so that each SILVA level contained multiple taxonomic levels. Therefore, the levels chosen (L12, 10, 8, 6, 4, and 3) generally represent summaries at progressively higher levels of taxonomy, but are not strictly adhering to species, genus and family level across each major eukaryotic group.

### 2.3. Model Testing

Postmortem interval prediction models were generated using Random Forest regressors based on sequence variant and taxa abundance data. Data were divided into subsets by sample type and normalized using the Calour library [28]. Using the Calour library, we chose to utilize total-sum scaling normalization, as opposed to rarefaction, to avoid the loss of statistical power by discarding reads and/or samples. Random Forest is insensitive to the methods of normalization used. For human body decomposition, each subset was partitioned based on individual for cross-validation so that the samples from the same individuals are either in the training set or testing set, but not both. Training refers to fitting or building the model while testing is equivalent to predicting. The accuracy of the models was measured using the mean absolute error (MAE), calculated as the deviation of the predicted from observed values and representing the average prediction error in the same unit of the original data. Within each dataset of each study, the best Random Forest regression model after hyperparameter tuning through cross-validation was selected to represent the final model. We also applied the model trained from one study to predict PMI of another study (i.e., cross-study prediction) to test the generalizability of the model. Each experiment was conducted over a different number of sampling days ranging from a total of 48 to 142 days (Table 1), so for consistency one model time frame was selected for inclusion in the model. Preliminary model tests were conducted to determine the time frame for use in this experiment, results of this analysis are presented in Figure S1. Overall, the inclusion of all experimental sampling days resulted in the highest MAE, while using only the first 25 days resulted in invariably lower MAEs (Figure S2). Therefore, data subset to the first 25 days of decomposition were selected for the modeling in this study. The modeling was done with Python machine learning package scikit-learn v19.0 [29]. Data were analyzed and graphics were generated using R software, version 3.4.1, the ggplot2 package, and matplotlib 2.0.0 [30,31,32]. We provide jupyter notebooks to enable reproduction of all modeling results as supplemental material.

## 3. Results

### 3.1. Cross-validation Error Rates

#### 3.1.1. Comparison of Sample Types

The datasets used for this study contained a variety of sampling sites, and those used most consistently were selected for this study to determine the best sampling location for microbiome prediction. Sample types investigated included cadaver-associated gravesoil, skin of the torso, and skin of the head. Results are summarized in Table 2. Both mouse model laboratory studies resulted in lower within study errors than the human studies. This is likely because these mouse studies were conducted under controlled laboratory conditions, as opposed to the human studies, which were conducted in the field with no control over environmental factors such as rainfall, temperature, and insect colonization. Overall, there is not a clear trend across the studies or gene markers of which sample type performed best. The lowest mean absolute error was from 16S rRNA data in gravesoil samples in a mouse decomposition study (mdc2), in which mice used were of the same breed, age, and were co-housed before being sacrificed for the study [11]. Within the two human studies, skin locations provided the lowest error for 16S rRNA marker, while soils provided the lowest errors for both microbial eukaryotic markers.

#### 3.1.2. Comparison of Genetic Markers across Sequence Variant and Taxonomic Levels

**Figure 1:**
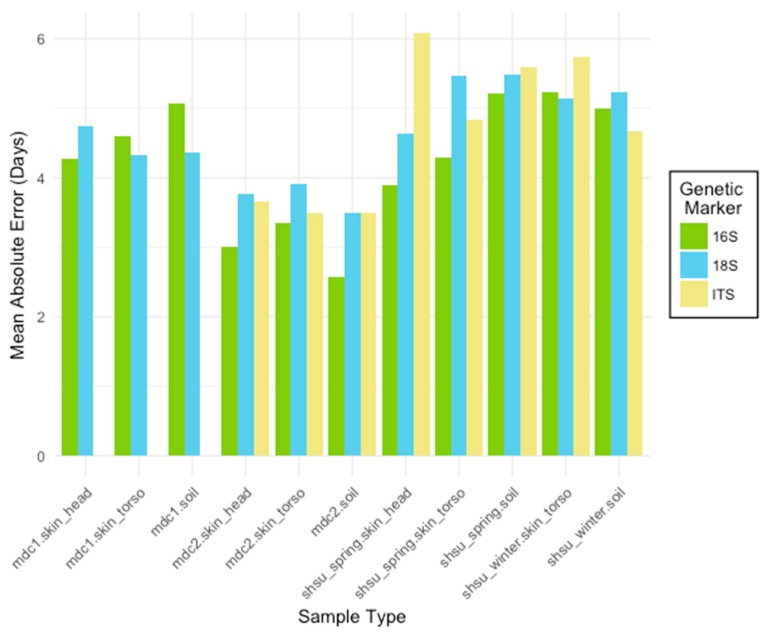
The mean absolute error (MAE) rates for Random Forest models trained to predict the postmortem interval (PMI). For each marker type (16S bacterial and archaeal ribosomal RNA (rRNA), 18S microbial eukaryote rRNA, internal transcribed spacer regions (ITS) fungal gene marker), models were generated for three sample types (skin_head, skin_torso, soil) from four studies (mouse decomposition 1 (mdc1), mouse decomposition 2 (mdc2), Sam Houston State University (SHSU) human April (shsu_spring), SHSU human February (shsu_winter)). Skin_head samples were not collected for shsu_winter. Datasets were subset to include only the first 25 sampling days. Though all marker types performed well, the 16S rRNA marker generally resulted in the most accurate PMI prediction models.

Three taxonomically-informative microbial gene markers (16S rRNA, 18S rRNA, and ITS) were compared at different sequence variant and taxonomic levels across sample types for each experiment (Figure 1, Table 2). Overall, the lowest within-study MAE for each experiment was generated using the bacterial and archaeal data (16S rRNA marker). However, all markers performed reasonably well with ITS producing the highest errors, which were as low as ±2.6 days over 25 days for mouse decomposition experiment 2 (mdc2). Within experiment, MAEs for each marker type were similar. Furthermore, models consistently performed best at the class and phylum taxonomic levels for 16S rRNA and 18S rRNA, and at the class level for ITS. The sequence variant level (highest resolution possible) had the highest MAEs compared to sequences summarized into lower levels of taxonomy.

### 3.2. Cross-study Error Rates

Cross-study error rates were generated between the two studies using human cadavers (Sam Houston State University (SHSU) spring and SHSU winter). Models were constructed for two sample types, cadaver-associated gravesoil and skin of the torso (skin), for each experiment, then tested on the same sample type for the other season. The skin of the head samples was excluded from this analysis as only one of the human datasets included this sample location. The postmortem interval was represented as 0 °C base accumulated degree day (ADD) to account for the differences in temperature between the two seasons. Resulting cross-study MAE are presented in Table 3, and plots of observed versus predicted PMI are presented in supplementary Figures S3–S9. For each marker type, the lowest error was generated using a gravesoil data set. The overall lowest cross-experiment error was generated from the model trained on the spring soil data set using bacterial and archaeal data at the phylum level.

Similar to the within-study errors, lower-level taxonomies generally resulted in more accurate models compared to sequence variant-level resolution. In particular, phylum level taxonomy appeared to provide the most accurate models overall in cross-experiment model testing. Mean absolute error was lowest at the phylum level for all three markers—48.686, 50.082, and 58.359 for 16S rRNA, 18S rRNA, and ITS, respectively, or approximately 5–6 chronological days. For models trained on the winter data set, the soil microbial eukaryotic 18S rRNA marker returned the lowest error. Internal transcribed spacer regions data resulted in the highest error in spring-trained data, while 16S rRNA data resulted in the highest error in winter-trained data. 

Overall, the models built on the spring data were more accurate in predicting the PMI of the winter data. This is likely because the spring data set spans a broader range of ADDs, which results in a more accurate model compared to the winter data set. Models trained on the winter skin samples resulted in the highest error when tested on the spring skin samples.

### 3.3. Important Feature Taxa

**Figure 2:**
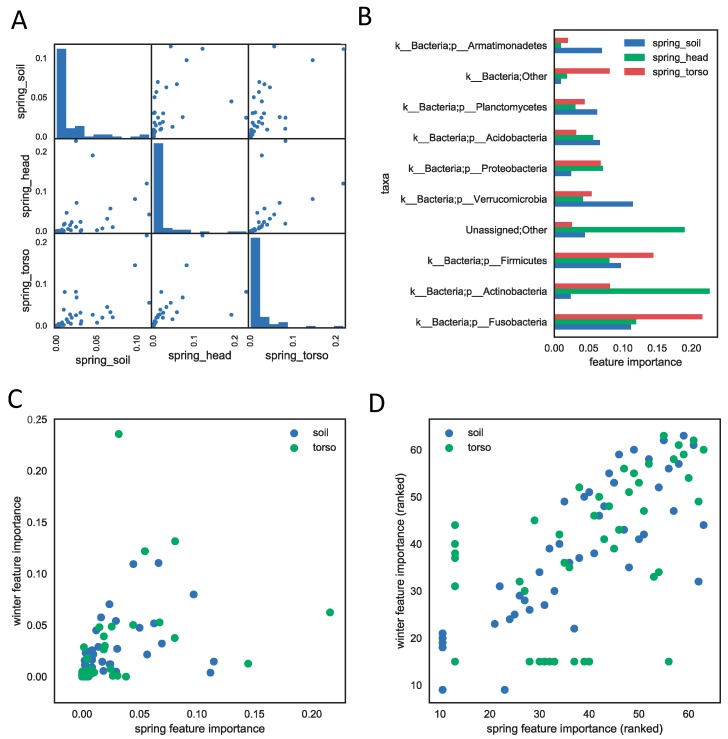
The feature importance measures the contribution of each phylum to the PMI regression model (results from SHSU spring study) using the 16S rRNA genetic marker. (**A**) The feature importance is correlated across three sample types. Each scatter plot shows the correlation between feature importances of every pair of models built from each sample type. Each dot represents a phylum and its value on the *x*- or *y*-axis represents its feature importance in the two models of sample types. The Spearman correlation coefficients are 0.90 (head vs. torso), 0.84 (head vs. soil), and 0.93 (torso vs. soil), with *p*-values < 0.01. The diagonal histogram plots show that most of phyla do not contribute much to regression models of each sample type. (**B**) The top ten phyla that are most informative for PMI prediction within each sample type. (**C**) The importance of the phyla to the regression models are highly correlated across spring and winter seasons. Each dot represents the importance of a phylum in winter season (*y*-axis) and in spring (*x*-axis). The correlation coefficients between winter and spring feature importances are 0.78 (soil) and 0.93 (torso), with *p*-values < 0.01. (**D**) same plot as (**C**), except axes are feature importance ranks instead of scores.

Not all taxa or taxa groups contribute temporal information equally. We assessed how informative each taxon was in the regression model by computing the average decrease of impurity during the tree splitting process in model training as one taxa or taxa group was removed iteratively for 16S rRNA data [20]. We reported the feature importance of phyla for all three sample types (soil, skin of the torso, skin of the head) in the human decomposition data sets (Figure 2). The importance of each phylum is highly correlated between sample types within study for both the spring and winter season (Figure 2A, Figure S10-winter). The soil and skin of the head samples appear to share the most bacterial phyla compared to the skin of the torso, and are the most highly correlated, with a Spearman correlation coefficient of 0.90 within spring samples. Samples from the skin of the head were not taken during the winter months for comparison, but the correlation between the spring skin of the head and winter soil was lower (0.77), though it is not clear whether the lower correlation was due to the sample type or the difference in season. Furthermore, only a few phyla contribute substantially to the models (Figure 2A). The most informative phyla for the spring season include Fusobacteria, Actinobacteria, Firmicutes, Verrucomicrobia, Proteobacteria, Acidobacteria, and Planctomycetes (Figure 2B). Furthermore, phyla important in the model were highly correlated between the spring and winter seasons (Figure 2C) of human decomposition (*p*-value < 0.01). 

## 4. Discussion

Machine learning methods are powerful tools for utilizing high dimensional datasets for prediction. Machine learning is ideal for finding patterns in complex and diverse microbiome data sets, and utilizing these patterns to predict outcomes [19], such as disease states [19,33]. Leveraging biological information associated with crime scenes is another excellent opportunity for the development of machine learning tools that utilize microbiome datasets. With the goal of developing the most robust model, we assessed how several variables affect within-study and cross-experiment errors for estimating PMI. Results of this meta-analysis indicate that the most robust models predicting PMI utilized cadaver-associated soil or skin data and the 16S rRNA gene marker summarized at class—or phylum—level taxonomies.

In this study, models for estimating PMI were developed using data from the first 25 sample days of each study, as preliminary data indicated the earlier sampling days resulted in more accurate models. Early decomposition may be the most accurate time frame because microbial succession is rapid and diversity is high compared to later stages of decomposition [10,11,16]. However, in the studies incorporated into this analysis the sampling was more frequent during early decomposition, and more frequent sampling has been demonstrated previously to improve model accuracy [11]. Therefore, the lower errors are likely, at least to some extent, an artifact of the change in sampling rate. Further investigation into the accuracy of models across different time frames is warranted.

Cadaver-associated soils as well as cadaver skin sites both appear to be promising sampling locations for developing microbiome-based PMI estimation tools. A wide diversity of sample sites has been investigated for microbial succession during decomposition, though few employed machine learning to estimate PMI, possibly due to small sample sizes. Soils and skin are both attractive sampling locations because they are easy to access without disturbing the remains. The soil microbiome has been shown to change predictably during mammalian decomposition, a change which is little affected by body mass [16,18] and soil type [11]. The skin, at various sample sites, has been demonstrated to accurately predict PMI using machine learning techniques [12,15,16]. Johnson et al. [15] demonstrated low errors for skin samples collected from the inner ear of human cadavers. Therefore, a comparison of different skin locations within a study would be useful to help identify locations in which microbial succession is the most clock-like. In our meta-analysis, we discovered fairly similar prediction accuracies using soils and skin, which may be because the two sample types are not independent. For example, Cobaugh et al. [16] demonstrated that the microbiomes associated with the skin of the body may transfer to the soil and persist in the soil microbiome.

Most microbiome studies, including those investigating the PMI, utilized the 16S rRNA gene marker. We have investigated the use of two additional microbial markers: 18S rRNA which amplifies microbial eukaryotes, and ITS, which amplifies fungi specifically. Although 16S rRNA provided the most accurate within-study models, the 18S rRNA marker had similar accuracies, and was more accurate in several cases during the cross-experiment validation. However, larger sample sizes would make these analyses considerably more robust. 18S rRNA has been previously shown to be more stable across seasons than bacteria [17], which may explain why it is robust in our cross-experiment model testing in which models were tested across the winter and spring seasons. Furthermore, for each marker gene, we tested multiple taxa levels and discovered that in all cases lower levels of taxonomy, particularly class and phylum levels, produced more accurate models, which agrees with results reported by Johnson et al. [15] on an independent data set of human cadaver skin samples. Furthermore, these models generally improve on error rates published in the original articles. For example, the mdc2 study originally reported error rates for 16S rRNA of 2.5 days (at the OTU level), and here we report 1.7 days at the Phylum level. However, we note that results reported here are not directly comparable to published results because we used a different processing method (e.g., deblur instead of 97% OTU clustering methods). Finally, we discovered that only a subset of phyla was highly informative to models and these important groups of microbes were similar across seasons, at least in one study. Those highly informative phyla are consistent with those reported in other studies [12,15]. This suggests that accurate models of the PMI may be constructed based on a subset of the microbial community, which may open the door for cheaper, targeted assays. 

In this study, we focused on utilizing a very powerful tool, Random Forests regression models. Every regression method has caveats. For example, Random Forest does not perform as well at the extreme ends of PMI. Another popular regressor, K-nearest neighbor (KNN) e.g., Johnson et al. [15], is a simple and intuitive model that works well on pattern recognition problems, but has a disadvantage in that distances between a given sample and all training data for each prediction must be computed, and thus it is less scalable to large data sets [34]. Linear regression (and its variants, lasso, ridge, elastic net) is also popular in regression analysis because it is possible to interpret how much every feature contributes to the model; however, the strong assumption of linearity between outcome and features are often violated. Support vector machine (SVM) is also a proved accurate method in many scenarios and handles high dimensional data very well, but it can perform poorly if there are many irrelevant features [34]. Although beyond the scope of this current article, a systematic comparison of regression methods will be informative and is planned as part of future research on a forthcoming large data set.

Determining timelines has been described as the Achilles heel of forensic pathology [35]. There are very few tools, and most are only applicable within the initial hours and days following death, and each method is vulnerable to biases [36]. Therefore, developing new tools that leverage independent information for estimating the time since death is critical. There is evidence that gene-meter expression data may be used to predict PMI. In a proof of principle study authors demonstrated that gene transcripts could be used to produce linear models of PMI with correlation coefficients of 1 [37]. This method may be an interesting alternative to microbial and entomological prediction methods as analyses are expanded. However, this has yet to be applied to human decomposition for further viability testing, and machine learning techniques were not applied.

The results of the current meta-analysis provide directions for future research on developing microbial-based models for estimating PMI. Currently, the greatest barrier to creating generalizable microbial models for estimating PMI is a lack of human cadaver-associated data sets from different environments and seasons. However, coordinated research is underway to overcome this limitation and generate a comprehensive data set to train, test, and generate robust models with larger sample sizes. Once available, existing and new datasets can be combined to determine the best generalizable model for estimating PMI based on microbes.

## Figures and Tables

**Table 1:** A summary of all studies included in the meta-analysis. Studies were obtained from the QIITA open source microbiome study management platform [22].

QIITA Study Number	QIITA Study Name	Our Study Name	Shorthand Name	Prep Number	Marker	Trim Length	OTU Table Type	Number of Days Sampled
714	A microbial clock provides an accurate estimate of the postmortem interval in a mouse model system	Mouse Decomposition 1	mdc1	769	16S	90 bp	reference-hit.biom	48
1889	A microbial clock provides an accurate estimate of the postmortem interval in a mouse model system—18S	Mouse Decomposition 1	mdc1	1204	18S	90 bp	all.biom	48
10141	Metcalf microbial community assembly and metabolic function during mammalian corpse decomposition	Mouse Decomposition 2	mdc2	1265	16S	90 bp	reference-hit.biom	70
				1038	18S	90 bp	all.biom	70
				345	ITS	100 bp	all.biom	70
10142	Metcalf microbial community assembly and metabolic function during mammalian corpse decomposition Sam Houston State University (SHSU) winter	SHSU Winter	shsu_winter	333	16S	90 bp	reference-hit.biom	132
				1166	18S	90 bp	all.biom	132
				335	ITS	100 bp	all.biom	132
10143	Metcalf microbial community assembly and metabolic function during mammalian corpse decomposition Sam Houston State University (SHSU) April 2012 exp.	SHSU Spring	shsu_spring	1107	16S	90 bp	reference-hit.biom	82
				1109	18S	90 bp	all.biom	82
				1110	ITS	100 bp	all.biom	82

All studies were downloaded as deblur processed tables along with the corresponding metadata information. Different table types and trim lengths were selected based on the availability and the marker type. 16S: 16S ribosomal RNA; 18S: 18S ribosomal RNA; ITS: internal transcribed spacer regions; OTU: operational taxonomic units.

**Table 2:** A comparison of the mean absolute error (MAE) of models built using data from each gene marker (16S rRNA, 18S rRNA, ITS) for each sample type (soil, skin_torso, skin_head).

Genomic Marker	Study Name	Sample Type	Sequence Variants	Species Level	Genus Level	Family Level	Order Level	Class Level	Phylum Level
16S	mdc1	soil	5.068	4.528	4.439	4.574	4.596	4.308	4.565
		skin_torso	4.602	3.744	3.577	3.353	3.889	4.377	4.070
		skin_head	4.272	3.816	3.816	3.747	3.442	**3.315**	4.672
	mdc2	soil	2.571	1.943	1.955	1.911	2.062	1.971	**1.737**
		skin_torso	3.357	2.926	2.898	2.783	2.826	2.942	2.856
		skin_head	3.001	2.383	2.379	2.340	2.467	2.369	2.405
	shsu_spring	soil	5.225	3.594	3.632	3.660	3.966	3.868	3.877
		skin_torso	4.303	3.830	3.807	4.106	4.343	4.311	4.022
		skin_head	3.890	3.506	3.385	3.577	3.342	**2.940**	3.006
	shsu_winter	soil	4.985	3.922	3.980	3.947	3.848	4.026	3.783
		skin_torso	5.237	4.543	4.483	4.385	3.970	3.704	**3.265**
18S	mdc1	soil	4.370	3.125	3.072	3.135	2.813	2.942	2.733
		skin_torso	4.333	3.821	3.447	3.030	3.549	**2.702**	4.521
		skin_head	4.744	4.583	4.138	4.616	4.251	3.775	4.657
	mdc2	soil	3.505	3.237	3.208	3.107	3.221	**3.043**	3.330
		skin_torso	3.907	3.870	3.856	3.676	3.910	3.867	3.704
		skin_head	3.772	3.761	3.575	3.725	3.665	3.819	3.912
	shsu_spring	soil	5.486	4.654	4.459	4.283	3.837	3.400	**3.264**
		skin_torso	5.457	4.654	5.196	5.404	5.264	5.754	5.974
		skin_head	4.645	4.571	4.370	5.148	5.028	4.763	5.218
	shsu_winter	soil	5.239	4.429	4.442	4.239	4.042	**3.449**	3.504
		skin_torso	5.141	4.880	4.721	4.962	5.028	4.660	4.604
ITS	mdc2	soil	3.497	3.169	3.157	2.957	2.941	2.820	2.797
		skin_torso	3.505	3.237	3.211	3.083	3.023	**2.597**	3.036
		skin_head	3.648	3.561	3.523	3.483	3.509	3.413	3.305
	shsu_spring	soil	5.586	4.735	4.836	4.629	4.980	**4.461**	4.713
		skin_torso	4.837	4.671	4.563	4.688	4.786	4.860	5.500
		skin_head	6.080	5.996	6.083	5.803	6.090	5.965	5.416
	shsu_winter	soil	4.675	4.114	3.965	3.954	3.933	**3.671**	4.077
		skin_torso	5.726	5.702	5.662	5.608	5.565	5.575	5.610

Data were collected from four studies (mouse decomposition 1 (mdc1), mouse decomposition 2 (mdc2), Sam Houston State University (SHSU) human April (shsu_spring), SHSU human February (shsu_winter)). The ITS marker was not sequenced for mdc1. Models were generated based on data from the first 25 days of decomposition and the model with the best MAE (days if decomposition) after parameter tuning was selected. The lowest error within each marker for each experiment is highlighted in bold, black text.

**Table 3:** The MAE of models used in cross-experiment testing using accumulated degree days (ADD) with a minimum developmental threshold of 0 °C.

Genomic Marker	Training Dataset	Sample Type	Sequence Variants MAE	Species Level MAE	Genus Level MAE	Family Level MAE	Order Level MAE	Class Level MAE	Phylum Level MAE
16S	Spring	soil	88.693	57.929	59.251	57.045	56.936	55.367	**48.686**
		skin	92.598	90.197	90.584	104.770	109.412	117.672	135.749
	Winter	soil	109.482	91.406	91.295	91.857	91.025	88.849	**83.312**
		skin	120.764	129.695	130.763	122.418	124.701	123.043	108.737
18S	Spring	soil	81.013	62.572	62.850	55.648	51.316	51.481	**50.082**
		skin	88.155	93.173	85.754	89.676	91.793	72.846	67.242
	Winter	soil	96.145	82.780	75.628	72.228	67.725	71.757	**63.465**
		skin	111.004	110.772	101.268	101.222	101.524	107.409	105.248
ITS	Spring	soil	111.806	94.797	94.742	93.504	85.282	80.856	**58.359**
		skin	101.852	96.815	99.272	101.086	104.162	106.043	94.468
	Winter	soil	104.775	99.360	96.392	96.604	93.564	87.709	**81.623**
		skin	114.027	110.865	107.026	113.294	117.302	115.937	87.274

Models were built using 16S rRNA marker human cadaver decomposition data from two seasons: spring and winter. Models were built on sequence variants data and family level, genus level, and species level taxonomy. Following model construction, the model was tested on the other dataset to evaluate the ability of the model to predict postmortem interval (PMI) beyond the original dataset. The lowest error for each marker within each cross-experiment test is in bold and black font.

## Supplementary Materials

The following are available online at http://www.mdpi.com/2073-4425/9/2/104/s1, Figure S1: Plots of the Mean Absolute Error (MAE) for models generated from three marker types of four datasets subset by sample type, further subset to 25, 50, and all sampling days; Figure S2: Plots of the Mean Absolute Error (MAE) for models generated on subsets of the data containing only the first 25 sampling days or sampling days 26–50; Figure S3: Random Forest models built from 16S, 18S, and ITS phylum data; Figure S4: Random Forest models built from 16S, 18S, and ITS class data; Figure S5: Random Forest models built from 16S, 18S, and ITS order data; Figure S6: Random Forest models built from 16S, 18S, and ITS family data; Figure S7: Random Forest models built from 16S, 18S, and ITS genus data; Figure S8: Random Forest models built from 16S, 18S, and ITS species data; Figure S9: Random Forest models built from 16S, 18S, and ITS sequence variant data; Figure S10: The feature importance of the phyla that measures the contribution of each phylum to the PMI regression model (results from SHSU winter study). QIIME further processing notes and jupyter notebooks describing the models are also included.
